# Acute Influenza B Infection Presenting as Cardiac Tamponade: A Case Report

**DOI:** 10.7759/cureus.11799

**Published:** 2020-11-30

**Authors:** Angelos Arfaras-Melainis, Hernando Cordero, Aditya Goyal, Linda Benes, Reka Salgunan

**Affiliations:** 1 Internal Medicine, Albert Einstein College of Medicine, Jacobi Medical Center, Bronx, USA; 2 Cardiology, Attikon University Hospital, Athens, GRC; 3 Pulmonary and Critical Care Medicine, Albert Einstein College of Medicine, Montefiore Medical Center, Bronx, USA; 4 Pulmonary and Critical Care Medicine, Albert Einstein College of Medicine, Jacobi Medical Center, Bronx, USA

**Keywords:** pericardial tamponade, cardiac tamponade, tamponade, influenza, influenza b, pericardial effusion, pocus

## Abstract

Influenza A and B acute infections usually affect primarily the respiratory system. In rare cases, however, the cardiovascular system is also compromised either via the direct effect of the virus or via the worsening of preexisting cardiac conditions. We present a rare case of acute Influenza B infection presenting as pericardial effusion and cardiac tamponade.

A healthy 32-year-old female was presented to the emergency room with influenza-like symptoms for four days, where she was monitored for a few hours and was subsequently discharged to home after testing positive for Influenza B by polymerase chain reaction (PCR). On the fifth day, she returned to the emergency room with worsening symptoms, primarily exertional dyspnea. She was hypotensive and tachycardic and temporarily improved with fluid administration. She was transferred to the intensive care unit, where a bedside point of care ultrasound (POCUS) and later a formal transthoracic echocardiogram revealed that she had pericardial effusion with sonographic signs of cardiac tamponade. Emergent pericardiocentesis was performed and resulted in hemodynamic and symptomatic improvement. The pericardial drain that was initially left in place and continued to drain pericardial fluid (700 ccs in total), was removed 3 days later, after echocardiographic confirmation of the resolution of the pericardial effusion. She completed a five-day course of Oseltamivir and was subsequently discharged home safely.

In summary, our case describes an acute Influenza B infection that was complicated by pericardial effusion and cardiac tamponade. It also highlights the importance of bedside POCUS and echocardiography in the early diagnosis and treatment of cardiac tamponade cases, frequently with pericardiocentesis as in our case.

## Introduction

Influenza A and B viruses affect the upper and lower respiratory tract, leading to increased morbidity and mortality every year which is reflected in the healthcare systems across the globe. As reflected by Center for Disease Control (CDC) [1] data from the past decade, the absolute number of hospitalizations for acute influenza infection range from 140,000 to 710,000, with mortality ranging from 12,000 to 56,000 deaths every year [1]. The most common presentations of Influenza include upper respiratory tract symptoms like rhinorrhea, nasal congestion, dry cough, scleral injection along with systemic symptoms like generalized malaise, fever, headache, and myalgias [2]. While cardiovascular complications of Influenza infection are rare, a few studies have highlighted an association between influenza and increased cardiovascular mortality largely attributed to an increased incidence of coronary events like acute coronary syndrome (ACS) and ST-segment elevation myocardial infarctions (STEMIs), particularly during the flu season [3,4]. Primary cardiac pathologies such as myocarditis, pericarditis, pericardial effusion, and cardiac tamponade caused by Influenza are not well-defined [3]. In this case report, we present a case of an otherwise healthy young female who presented with cardiac tamponade in the setting of acute Influenza B infection.

## Case presentation

**Figure 1:**
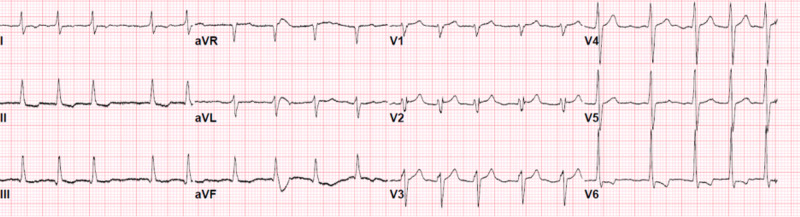
Electrocardiogram showing sinus tachycardia with decreased QRS voltage

A 32-year-old female with no known past medical history, no recent travel, working as a home aid, presented to our emergency department at the end of January 2020, with intermittent fevers, generalized body aches, non-productive cough, congestion, and nausea for four days. She was found to be positive for Influenza B by polymerase chain reaction (PCR). After a few hours of monitoring in the ED, she was noted to tolerate oral intake, had stable vital signs, and was subsequently discharged home. However, she returned to the ED the next day with worsening symptoms. She reported that although her cough had subsided, she continued having worsening myalgia and developed new epigastric pain, orthopnea, and foul-smelling urine. She denied chest pain, palpitations, lower extremity edema, or vomiting at the time. Upon presentation to the ED, she was found to be hypotensive with systolic blood pressure (BP) in the 80’s, tachycardic with an initial heart rate (HR) of 180 bpm, and afebrile. Her hemodynamic status improved after she received 1 L of Ringer’s lactate; BP increased to 90s/60s, HR decreased to 120s. Her initial physical examination was notable only for decreased breath sounds bilaterally at the bases. Her cardiac sounds were clear and regular without any murmurs, rubs, or gallops, and no jugular vein distention (JVD) was noted. The remaining physical examination including abdominal, neurological, and musculoskeletal was unremarkable. Initial lab work revealed no leukocytosis, anemia, or electrolyte abnormalities. Cardiac enzymes were negative; however, lactate was elevated to 4.2 mmol/L. Urinalysis showed moderate proteinuria, positive leukocyte esterase, nitrites, and elevated white blood cell count (WBC) concerning urinary tract infection. Chest X-ray revealed mild prominence of the right mediastinal contour at the area of the superior vena cava (SVC). Her initial electrocardiogram (EKG) showed sinus tachycardia with decreased QRS voltage (Figure 1).

**Figure 2:**
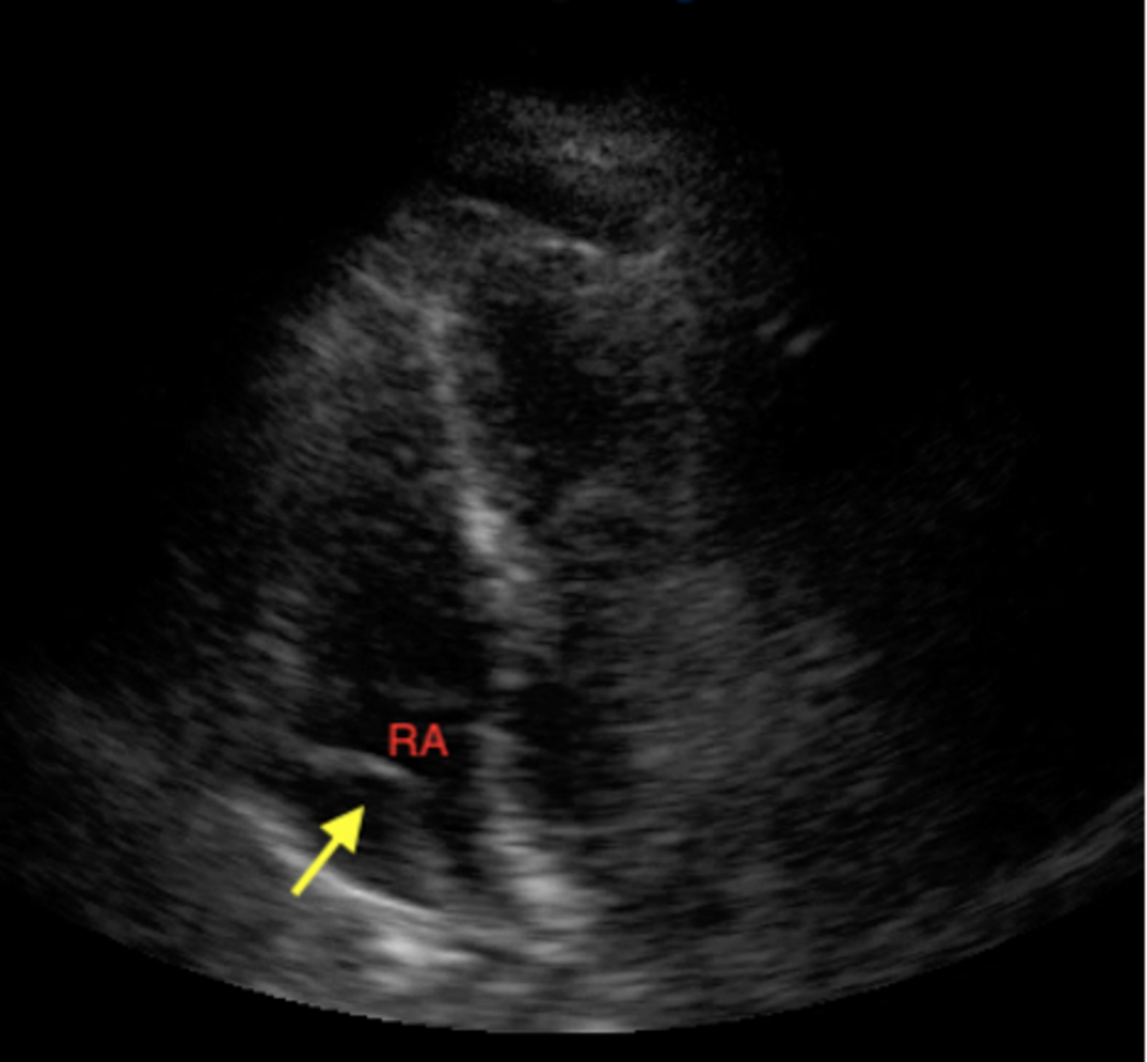
POCUS showing the right atrial diastolic collapse

**Figure 3:**
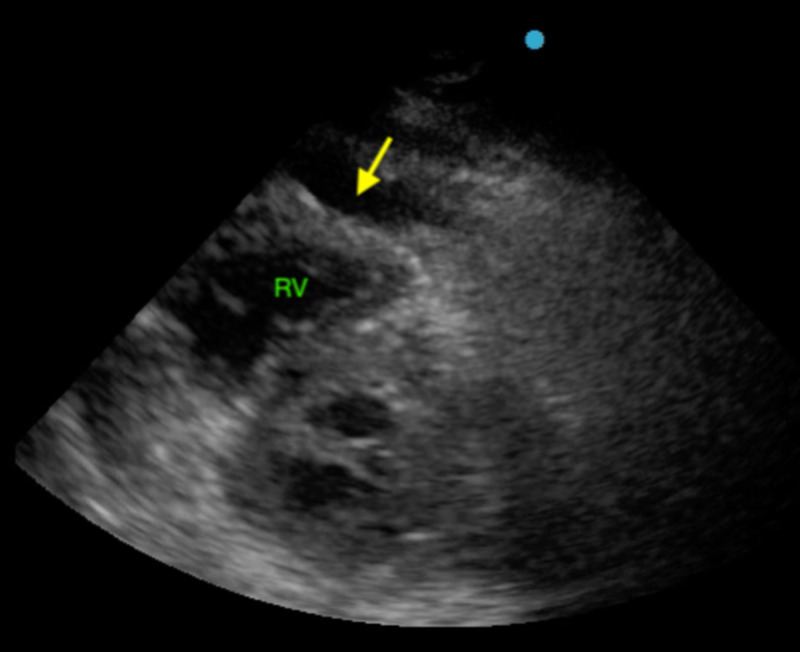
POCUS showing the right ventricular diastolic collapse

**Figure 4:**
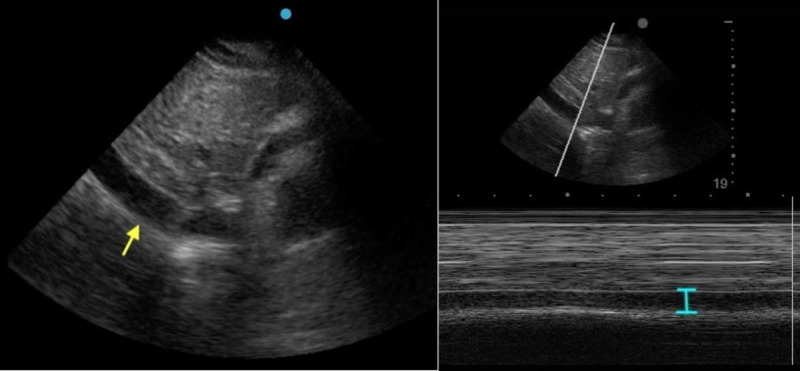
POCUS showing the increased diameter of inferior vena cava

Point of care ultrasound (POCUS) showed moderate pericardial effusion with signs of right atrial (RA; Figure 2) and right ventricular (RV; Figure 3) diastolic collapse along with increased inferior vena cava (IVC) diameter without respirophasic variation (Figure 4).

The patient was admitted to the medical intensive care unit (MICU) and a subsequent formal echocardiogram confirmed the presence of tamponade physiology. The patient underwent emergent pericardiocentesis in the catheterization laboratory where 250 cc yellow-colored pericardial fluid was removed with immediate symptomatic and hemodynamic improvement. A pericardial drain was placed and collected a total of an additional 450 ccs in the next 72 hours. After removal of the pericardial drain on the fourth day of admission, a repeat transthoracic echocardiography (TTE) confirmed no re-accumulation of pericardial fluid with the restoration of normal contractile physiology.

The patient also received a five-day course of Oseltamivir and a three-day course of antibiotics for urinary tract infection (urine culture grew pansensitive *Escherichia coli*, blood cultures remained negative) during her hospital stay. By the fifth day of admission, the patient reported feeling back to baseline, asymptomatic, without any evidence of residual pericardial effusion and was, therefore, discharged home.

## Discussion

Herein, we highlight a rare potential cardiac complication of Influenza B. A thorough evaluation of all possible potential causes of pericardial tamponade was done during the initial evaluation (Table 1).

**Table 1:** Alternate diagnosis for the pericardial effusion and respective test results ft4: free thyroxine, TSH: thyroid-stimulating hormone, BUN: blood urea nitrogen, eGFR: effective glomerular filtration rate, ANA: antinuclear antibodies, ANCA: antineutrophilic cytoplasmic antibodies, C3: complement component 3, C4: complement component 4, ACS: acute coronary syndrome, PPD: purified protein derivative, Hep: hepatitis, RVP: respiratory viral panel.

Diagnosis	Laboratory test result
Hyperthyroidism	fT4 = 0.994 ng/dl, TSH = 5.03 U/L (wnl)
Hyperuricemia	BUN = 9 mg/dL, creatinine = 0.3 mg/dL, eGFR = 258 ml/min/1.73m^2^ (wnl)
Autoimmune disorders	ANA, ANCA were negative, C4 = 10.9 mg/dl (wnl) and C3 = 75.6 mg/dl (mildly decreased)
ACS	Cardiac enzymes negative
Tuberculosis	PPD and quantiferon were negative
Hepatitis	Hep B and C serologies were negative
Other viruses	RVP negative
Trauma	No evidence of trauma on imaging
Malignancy	No malignant cells in the pericardial fluid

Thyroid function abnormalities were ruled out by normal levels of TSH and fT4, uremia was ruled out by the patient’s normal kidney function (BUN = 9 mg/dL, Creatinine = 0.3 mg/dL, eGFR = 258 ml/min/1.73m^2^). Among inflammatory markers, erythrocyte sedimentation rate (ESR) was within normal limits; however, C-reactive protein (CRP) was significantly elevated (109.6 mg/L). As for rheumatological causes, the patient denied any personal or family history of autoimmune diseases, rashes, photosensitivity, or other signs and symptoms of SLE. ANA and ANCA were negative, C4 level was normal although, C3 level was mildly decreased (75.6 mg/dL). Additionally, given that patient was from Ecuador (endemic for TB) and had moved to New York four years ago, a PPD was placed and Quantiferon was sent both of which were negative. Of note, the patient’s albumin decreased during hospitalization from 4 g/dL to 2.6 g/dL. A respiratory viral panel was sent in addition to the influenza PCR and was otherwise unremarkable. Lastly, pericardial fluid studies were inconclusive as shown in Table 2. 

**Table 2:** Pericardial effusion studies LDH: lactate dehydrogenase, wbcs: white blood cells, rbcs: red blood cells.

Cytology	Negative
Culture and Gram stain	Negative
Albumin	<0.2 g/dL
Protein	<0.2 g/dL
LDH	<10 U/L
Cell count	wbcs = 277/ul, rbcs 250/ul, segmented neutrophil % = 32, lymphocyte % = 10, monocyte % = 57, eosinophil % = 1

Interestingly, initial POCUS also showed small bilateral pleural effusions with atelectatic lung and a small amount of intra-abdominal fluid (fluid was present in the splenorenal recess). We presumed that only one etiology could explain the aforementioned signs of generalized serositis in this patient and with the rest of the workup being negative, influenza B was identified as the most possible culprit.

Undoubtedly, Influenza is known to usually affect the respiratory system only sparing the cardiovascular system. However, adding such a case to the growing evidence of cardiovascular (CV) complications due to Influenza, the true incidence of myocardial and pericardial involvement could be underestimated. Cases of both myocarditis and pericardial effusion with or without tamponade physiology secondary to influenza have been reported [5-8] but in most cases of cardiac involvement, Influenza A was identified and not Influenza B [9,10] (as in our case).

As far as pericardial disease is concerned, it is worth mentioning that the key in the sequence of events leading to tamponade is the rate of fluid accumulation and not the volume itself. A rapid buildup of pericardial fluid in a relatively small and otherwise non-compliant pericardial space could lead to a large change in the intrapericardial pressure for a small change in the volume. As this intrapericardial pressure exceeds the intracardiac pressure, the hallmark features of tamponade, including pulsus paradoxus, RA and RV diastolic collapse [11,12] start to manifest. Following the above paradigm, the patient in our case who has no other obvious reason to have a chronic pericardial effusion based on her history and age could have developed a moderate effusion after being infected with the influenza B virus and that rapid accumulation led to tamponade and hemodynamic compromise.

Going with well-established treatment guidelines for pericardial tamponade with hemodynamic instability, our patient was promptly treated with pericardiocentesis and fluid removal [11,13]. Usually, this immediately decreases the intrapericardial pressure, allowing the RA and RV to fully expand, increasing the preload, thus improving the patient’s cardiac output and clinical symptoms, as it happened in the case of our patient. Other measures that are indicated as adjuncts to pericardiocentesis include adequate volume resuscitation, and the use of vaso-pressive agents if needed to maintain a mean arterial pressure (MAP) > 65 mmHg [13].

Regarding treatment for influenza, two agents, namely Oseltamivir and Zanamivir, have been proven to decrease the duration of symptoms by approximately one day, and thus, are recommended as first-line agents that should be initiated within 48 hours from symptom onset. No benefit was identified when these agents were used after the first 48-hour window in patients with uncomplicated influenza infections although, Oseltamivir was beneficial even when used after the first 48 hours in patients with severe symptoms that required hospitalization [14,15]. Unfortunately, there are no data regarding potential agents for prophylaxis and/or treatment of cardiac complications of influenza, other than general post-exposure prophylaxis. No studies have been performed to assess the effect of the above-mentioned antivirals on the prevention or treatment of myocarditis and pericardial effusion in patients with influenza.

## Conclusions

Our case highlights a rare manifestation of influenza infection: pericardial effusion and tamponade. It is also one of the rare cases in the literature of influenza B specifically being associated with a cardiac complication, as influenza A is much more commonly identified as a culprit in similar cases. It is of utmost importance to keep a low threshold to evaluate for pericardial effusion in patients with influenza presenting with concomitant orthopnea and hypotension. Implementation of POCUS for early diagnosis of potentially life-threatening entities such as pericardial effusion and tamponade could serve as an invaluable tool in the diagnosis and prompt treatment leading to favorable outcomes.
